# Repeated Evolution of Testis-Specific New Genes: The Case of Telomere-Capping Genes in *Drosophila*

**DOI:** 10.1155/2012/708980

**Published:** 2012-07-11

**Authors:** Raphaëlle Dubruille, Gabriel A. B. Marais, Benjamin Loppin

**Affiliations:** ^1^UMR 5534, Centre de Génétique et de Physiologie Moléculaire et Cellulaire, Centre National de la Recherche Scientifique, Université Claude Bernard Lyon 1, Université de Lyon, 69622 Villeurbanne, France; ^2^UMR 5558, Laboratoire de Biométrie et Biologie Evolutive, Centre National de la Recherche Scientifique, Université Claude Bernard Lyon 1, 69622 Villeurbanne, France

## Abstract

Comparative genome analysis has allowed the identification of various mechanisms involved in gene birth. However, understanding the evolutionary forces driving new gene origination still represents a major challenge. In particular, an intriguing and not yet fully understood trend has emerged from the study of new genes: many of them show a testis-specific expression pattern, which has remained poorly understood. Here we review the case of such a new gene, which involves a telomere-capping gene family in *Drosophila. hiphop* and its testis-specific paralog *K81* are critical for the protection of chromosome ends in somatic cells and male gametes, respectively. Two independent functional studies recently proposed that these genes evolved under a reproductive-subfunctionalization regime. The 2011 release of new *Drosophila* genome sequences from the *melanogaster* group of species allowed us to deepen our phylogenetic analysis of the *hiphop/K81* family. This work reveals an unsuspected dynamic of gene birth and death within the group, with recurrent duplication events through retroposition mechanisms. Finally, we discuss the plausibility of different evolutionary scenarios that could explain the diversification of this gene family.

## 1. Introduction

In the past decade, rapid progress has been made on the origin and evolution of new genes thanks to the genomics revolution [1]. Many cases of gene birth are now documented, and they have revealed that the mechanisms for new gene formation are surprisingly diverse. They include DNA-based duplication, RNA-based duplication (retroposition or retroduplication), gene fusion (chimerization), *de novo* gene origination, domestication of transposable elements, and horizontal gene transfer [1, 2]. Remarkably, many new genes show a male-biased expression and a majority of these are actually specifically expressed in the testis. Indeed, this organ seems to have a critical role in gene birth and evolution [1]. Two of the first documented cases of gene origination, *Pgk2* in mammals and *Jingwei *in *Drosophila,* are both testis-specific [3, 4]. More recent work on retroduplication showed an overall propensity of young retrogenes to be testis specific [5, 6]. Other types of new genes also tend to show testis-specificity or testis-biased transcription (e.g, [7–12]). 

Several explanations have been proposed for this tendency of new genes to be testis specific [1, 13, 14]. The first explanation relies on a peculiarity of sex chromosome biology called MSCI (male sex chromosomes inactivation). In mammals and *C. elegans, *the sex chromosomes are inactivated during male meiosis, probably as a consequence of a general mechanism to avoid recombination between nonhomologous sequences [15, 16]. MSCI is expected to drive genes expressed during male meiosis out of the sex chromosomes. This was observed by looking at retrogenes in mammals [17–19]. In mice, in particular, it has been shown that the X parental genes are ubiquitously expressed except in testis, and this is complemented by a testis-specific expression of their daughter autosomal retrocopies in agreement with the “escape from MSCI” hypothesis [19]. In *Drosophila*, the “exodus” of testis-specific genes out of X affects RNA-based [5] and possibly DNA-based duplicates [20]. However, the actual contribution of MSCI to this phenomenon and even the very existence of MSCI in *Drosophila* are actively debated issues [21–26]. 

Another hypothesis has been proposed to explain this above-mentioned pattern, especially in *Drosophila* where MSCI is controversial. It involves the interaction between dosage compensation (DC) and sex-biased expression [24, 27, 28]. The massive Y gene loss or silencing generates an imbalance of expression for X-linked genes compared to autosomal genes in males. DC mechanisms have evolved to even X and autosomal gene expression [29]. In mammals, where one X is inactivated in females, the X is hypertranscribed in both sexes, while in *Drosophila* the X is hypertranscribed in males [29, 30]. In *Drosophila*, male-biased genes have been shown to evolve mostly by hyperexpression in males only [28, 31]. However, such evolution of male-biased expression is difficult on the X chromosome because it is already hypertranscribed due to DC [24]. In agreement with this model, it has been shown that highly expressed male-biased genes are underrepresented on the X chromosome [28] and that dosage-compensated X genes tend to have autosomal retrocopies with male-biased expression [27]. 

However, the “escape from MSCI” and “escape from DC” hypotheses can only explain the evolution of new testis-specific genes involving the relocation out of the sex chromosomes but not those involving autosomes only. Another more general explanation has been recently proposed [13, 14]. In species with two sexes, mutations with sex antagonism (beneficial for one sex, deleterious for the other) can arise [32]. The presence of two sex-antagonistic alleles of a gene can cause an intralocus sexual antagonism [33]. Evolving sex-biased expression is a way to solve the conflict. However, this cannot work for housekeeping genes that need to be expressed in both sexes. In this case, duplication can resolve the intralocus sexual conflict, with the parental copy remaining expressed in both sexes and the new one being expressed only in one sex. Data in *Drosophila* suggests that testis is the tissue where sex antagonism is by far the strongest, and most male-biased genes are indeed expressed in testis [34]. In practice, solving intralocus sex conflict for housekeeping genes will imply getting a new copy expressed in testis [13, 14]. Gallach et al. [35] reported that 83% of the relocated copies of the mitochondrial genes found in the nuclear genome exhibit testis-specific expression. Importantly, about half of these relocation events involved autosomes only and could not be explained by the “escape from MSCI” and “escape from DC” hypotheses. dN/dS analysis of these genes suggested that the testis-specific copies tend to evolve under positive selection. Other examples of housekeeping genes show similar patterns, which fits well with the idea of resolving sexual conflict by duplication [13, 14, 36]. 

Finally, testis-specific new genes may be more common just because new genes arise more easily when expressed in the testis (the “out of testis” hypothesis, see [1]). In mammals, the chromatin in male germ cells is characterized by the presence of histone variants and histone marks favoring open chromatin, widespread demethylation of CpG-enriched promoters, and elevated levels of the transcription machinery components [37, 38]. Similarly, in *Drosophila* primary spermatocytes, very high level of transcriptional activity ensures the production of most mRNAs required for the postmeiotic differentiation program of male germ cells [2, 39]. This highly permissive state of chromatin as well as other peculiar features of male germ cells may have facilitated the expression of newly arisen genes in testis during their early evolution [40].

Many papers call for more functional studies of new genes. Here we review the case of the *hiphop*/*K81* telomere capping genes in *Drosophila*, for which detailed functional studies are available. We also present new results on the evolution of the *hiphop*/*K81* genes and discuss functional and evolutionary data with respect to the hypotheses presented above.

## 2. K81 as a Case of Reproductive Specialization of an Essential Telomere Protein

### 2.1. * Drosophila * Telomeres and Capping Proteins

Telomeres are essential structures at the end of eukaryotic chromosomes that are generally composed of highly repetitive DNA associated with specific proteins. The elongation of repetitive telomeric DNA counteracts the slow erosion of chromosome arms caused by the incomplete replication of DNA extremities at each S-phase. Telomere elongation is mediated in most eukaryotes by the conserved enzyme telomerase, a reverse transcriptase that adds small G-rich repeats, such as (TTAGGG)n, at the end of chromosomes. In addition, telomeres function as protective caps that prevent the recognition of chromosome ends as DNA double-strand breaks by the DNA repair machinery and their irreversible and deleterious ligation [41–43]. In most eukaryotes, this capping function is largely dependent on several DNA binding proteins that specifically recognize the small repeats added by the telomerase complex. *Drosophila* represents an exception in telomere biology as this model organism lacks telomerase. In this species, the “end replication problem” is solved in an original manner, by the controlled insertion of specialized telomeric retrotransposons at chromosome extremities [44]. Although repetitive by nature, *Drosophila* telomeric DNA thus lacks large arrays of small repeat motifs and associated binding proteins. Instead, the capping function of *Drosophila* telomeres is ensured by proteins that possess the remarkable ability to bind chromosome ends in a sequence-independent manner [45–47]. 

Well-characterized *Drosophila* capping proteins include HOAP, HP1a, Modigliani (Moi), Verrocchio (Ver), and HipHop [48–53]. Mutations affecting capping genes are all zygotic lethal and induce chromosome end-to-end fusions that are detectable in rapidly dividing cells. Telomere fusions form dicentric chromosomes that break in mitotic anaphase result in genomic instability. Despite their critical role for the maintenance of genome integrity, *Drosophila* capping proteins are rapidly evolving. With the exception of heterochromatin protein 1a (HP1a), which has additional functions in the nucleus, and possibly the OB-fold containing protein Ver, other capping proteins do not seem to have any ortholog in yeasts, mammals, or plants [46].

### 2.2. *K81*, a Male Germline Paralog of the HipHop Capping Protein

The *Drosophila ms(3)K81* (*K81*) gene was originally identified through a unique male sterile mutation found in a Japanese population of *D. melanogaster* [54]. *K81* mutant males produce apparently normal sperm that are capable of fertilizing eggs. However, the resulting embryos invariably die before hatching, a phenotype which actually makes *K81* one of the very rare paternal effect, embryonic lethal mutations. Furthermore, eggs fertilized by *K81* mutant sperm develop as nonviable, aneuploid, or haploid embryos, after the loss of paternal chromosomes during the first zygotic nuclear division [54–56]. Despite the critical requirement of K81 for the integration of paternal chromosomes into the diploid zygote, its molecular identification unexpectedly revealed a small, intronless gene, encoding a nonconserved protein [55]. In fact, the *K81* gene appeared restricted to the nine species comprising the *melanogaster* subgroup. Loppin et al. [55] also identified another gene paralogous to *K81*, now known as *Hiphop*, which was present in species of the *melanogaster* subgroup as well as in the more distantly related *D. pseudoobscura* genome. The conserved synteny around the *hiphop* locus in *D. melanogaster* and *D. pseudoobscura* strongly indicated that *hiphop* was the ancestor gene, while *K81* appeared after the duplication of *hiphop* at the root of the *melanogaster* subgroup. 

The *hiphop* gene is located in chromosome arm 3L and has a unique predicted intron immediately upstream its coding sequence. *hiphop* is expressed in most tissues at low to moderate levels, but it is also strongly transcribed in adult ovaries, suggesting that the HipHop protein is required during early embryo development. *hiphop* mutants are zygotic lethal and die in larval stages. In contrast to *hiphop*, *K81* expression is essentially restricted to the male germline [55], and adult flies homozygous for a *K81* null allele are viable. The *K81* gene (chromosome arm 3R) has no intron and presumably shares its 5′ regulatory sequences with its neighbor gene *Rb97D*, which is also strongly expressed in the testis. Taken together, these features fit well with a retroposition event at the origin of *K81* [55]. More recently, the independent findings that *hiphop* and *K81* encoded telomere capping proteins [57, 58] eventually provided the functional frame that was required to revisit the molecular evolution of these paralogs. 

## 3. Evolution of K81: Functional Innovation or Reproductive Specialization?

HipHop and K81 are small proteins (221 and 184 residues, resp.) that do not display any known domain or motif [55]. HipHop was originally implicated in telomere biology through its physical interaction with the HOAP and HP1a capping proteins [50]. Furthermore, knocking down *hiphop* in somatic cells induces telomere fusions at high frequency indicating that HipHop is critical for the capping of chromosome ends. Finally, the HipHop protein is specifically enriched at telomeres and this localization occurs independently of the DNA sequence [50]. Similarly, K81 was demonstrated to associate with telomeres in the male germline, in a way similar to HipHop in somatic cells. Indeed, functional GFP::K81 fusion protein was observed at telomeres in spermatocytes as well as in postmeiotic spermatids and in mature gametes [57–59]. In spermatids, GFP::K81 accumulates into a small number of foci (that presumably correspond to clustered telomeres) that also contain HOAP and HP1a, but not HipHop. During the condensation of spermatid nuclei in *Drosophila* as in many animals, histones are massively replaced by sperm-specific, nonhistone chromosomal proteins such as protamines [60]. Interestingly, in the absence of K81, HOAP and HP1a are not maintained at telomeres after the histone-to-protamine transition, suggesting that K81 is required for the stability of the capping complex in the peculiar chromatin environment of condensing spermatid nuclei [57]. Using the GFP::K81 transgene allowed to demonstrate that the K81 capping protein remains associated with paternal telomeres until zygote formation, where it is required for the protection of paternally-transmitted telomeres [57]. Accordingly, in eggs fertilized by *K81* sperm, paternal chromatin bridges resulting from telomere fusions are observed during the first mitosis [55, 57, 58]. After fertilization, maternally provided HipHop progressively replaces K81 at paternal telomeres, which is no longer detectable after two or three nuclear cycles. 

Why does *Drosophila melanogaster* require a second HipHop-related protein to protect telomeres in postmeiotic germ cells when other species outside the *melanogaster* subgroup only have a single *hiphop* gene? First, experimental evidence clearly indicates that HipHop is not capable to functionally replace K81 in the male germline. Indeed, a transgene expressing *hiphop* in male germ cells using the *K81* regulatory sequences cannot restore the fertility of *K81* mutant males [57, 58]. Interestingly however, *hiphop* is nevertheless capable of restoring HOAP and HP1a foci at telomeres in early spermatid nuclei, but all three capping proteins eventually disappear when histones are replaced with protamines [57]. Thus, these observations support the idea that K81 has become specialized in protecting telomeres in the highly peculiar chromatin environment of condensing spermatid nuclei. Second, and quite remarkably, the single HipHop-related protein of *D. virilis* (which lacks K81) was found associated with telomeres throughout spermiogenesis, strongly indicating that the ancestral *hiphop* gene in the *melanogaster* lineage was required to protect telomere in all cells, including male germ cells [58]. Taken together, these studies suggest a reproductive subfunctionalization by duplication-degeneration-complementation (DDC) (see [61]), in which the ancestral HipHop lost its ability to protect telomeres in postmeiotic germ cells after the gene duplication event. Meanwhile, the duplicated copy acquired male germline specific expression and specialized in the capping of telomeres in the peculiar sperm chromatin environment. This scenario is actually supported by the analysis of nonsynonymous and synonymous nucleotide substitutions of *hiphop* and *K81* sequences, which indicated that these genes evolved under purifying selection as in the typical DDC model [57].

## 4. Diversification of the HipHop Protein Family: The Rule or the Exception?

Based on the first available twelve *Drosophila* sequenced genomes, the *hiphop*/*K81* duplication appeared specific to the *melanogaster* subgroup of species. Notably, the *hiphop* gene was found at the same genomic position in all species of the *Sophophora* subgenus while *K81* was restricted to the *melanogaster* subgroup [55]. This view, however, was biased by the absence of sequenced genomes belonging to the other subgroups comprising the *melanogaster* group. Indeed, this large and complex group includes at least ten subgroups with many species that can be partitioned into three main phylogenetic clades [62]. Recently, the genome sequences of eight additional species representative of several other subgroups in all three clades were released by the modENCODE consortium (modencode.org) and were made available by Flybase (flybase.org).

**Figure 1:**
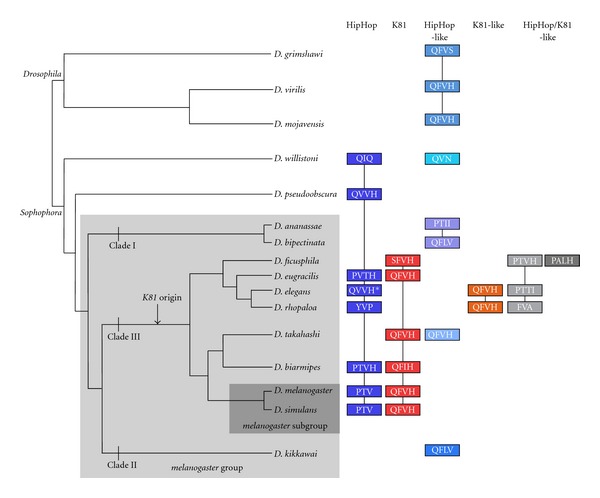
The HipHop/K81 protein family. A tree representing the schematic phylogeny of *Drosophila* species as described by Yang et al. [62]. For each species, the HipHop/K81-like proteins are represented as rectangles. These proteins were identified by tBLASTn search in Flybase (http://flybase.org/blast/). For the 8 new sequenced *Drosophila* genomes (*biarmipes, elegans, eugracilis, ficusphila, bipectinata, rhopaloa, takahashii,* and* kikkawai*), which are not yet annotated, an ORF corresponding to the protein was identified and used to determine the putative whole protein sequence (see also Table 1) except for *D. elegans*-HipHop due to poor sequence quality (∗). HipHop-like or K81-like proteins are proteins more closely related to HipHop or K81, respectively, whereas proteins whose phylogenetic origin was ambiguous HipHop or K81, are referred to as HipHop/K81-like proteins. For each protein, we identified the PTV or QFVH motif (see text) in the C-terminal domain that was described by Gao et al. [58] as responsible for the functional divergence between HipHop and K81 in sperm telomere protection. This PTV/ QFVH motif is indicated for each protein in the corresponding rectangle. BLAST analysis using 5 kbp upstream and downstream the *hiphop/K81*-like genes allowed to identify the orthologous region in the *melanogaster* genome. A same color code and a line connecting proteins indicate that the synteny block is conserved between the corresponding genes.

Interestingly, our combined BLAST analyses and microsynteny comparisons revealed an unsuspected diversification of the *hiphop* family in the *melanogaster* group (Figure 1). First, the *K81* gene is also present in species from four other subgroups belonging to clade III: *ficusphila*, *eugracilis*, *takahashi*, and *suzukii* (represented by *D. biarmipes*). Conversely, within the *melanogaster* group, *K81* is absent from the two available species from clade I (*D. ananassae* and *D. bipectinata*) and from the single representative species of clade II (*D. kikkawai*). Thus, *K81* appears to have a broader phylogenetic distribution than initially thought, and the gene duplication event at the origin of this gene probably occurred at the base of clade III (Figure 1).

We have also noticed the absence of *K81* in two species of clade III (*D. elegans* and *D. rhopaloa*) where it is presumably replaced by a paralog at another genomic position (*K81-like*, in orange in Figure 1). Interestingly, synteny block comparisons indicates that these two *K81-like* genes are apparently located on the X chromosome (Table 1), in contrast to the general tendency of testis-specific retrogenes to avoid the X [5, 20]. 

Finally, we observed that the original *hiphop* gene was independently lost or relocated at least at three occasions. In *D. bipectinata* and *D. ananassae* (clade I), *hiphop* is apparently replaced by a single *hiphop-like* paralog. In *D. ficusphila* and *D. takahashi* (clade III), *hiphop* is absent but one or two additional paralogs are present, in addition to the original *K81 *gene. Interestingly, one of the new paralogs found in species of clade III (represented in light gray in Figure 1) is conserved between *D. ficusphila*, *D. elegans*, and *D. rhopaloa*, but not in the *D. eugracilis* lineage. Thus, the repertoire of *hiphop/K81* related genes in the *Drosophila* group of species is extremely dynamic, with multiple gene gains and losses observed at several levels of this radiation. Some species have three members of this gene family in their genomes, while all other species have either one or two. The fact that at least one *hiphop*-related capping gene is present in all *Drosophila* genomes sampled so far underlines the essential role of these genes for telomere protection. Importantly, the tendency of *hiphop* to duplicate is not restricted to the *melanogaster* group since an independent duplication of this gene occurred in the lineage leading to *D. willistoni* (*willistoni* group) ([57]; Figure 1).

Based on a combination of K81/HipHop protein alignment and complementation tests with mutant proteins, Gao and colleagues [58] proposed that a small QFVH motif near the C-terminus of K81 is critical for the protection of telomeres in mature gametes. Interestingly, in HipHop proteins from the *melanogaster* subgroup, this motif is replaced with a PTV tripeptide which functions in somatic cells but not in mature male gametes. However, non-*melanogaster* species that harbor a single *hiphop*-related gene display a “male germline-like” motif which also begins with a glutamine residue as in QFVH [58]. The presence of such a motif is probably important for these proteins to fulfill their role in all cells, including postmeiotic male germ cells. We have extended this analysis to the new available members of the family and found that this tendency is generally confirmed for the additional proteins. For instance, the K81-like proteins from *D. elegans* and *D. rhopaloa* (Figure 1 and Table 1). In addition, the single HipHop-like proteins from *D. bipectinata* (clade I) and *D. kikkawai *(clade II) have also a motif of the male germline type (QFLV). The only exception is *D. ananassae* where the single HipHop protein is apparently of the somatic type (PTII). 

**Figure 2:**
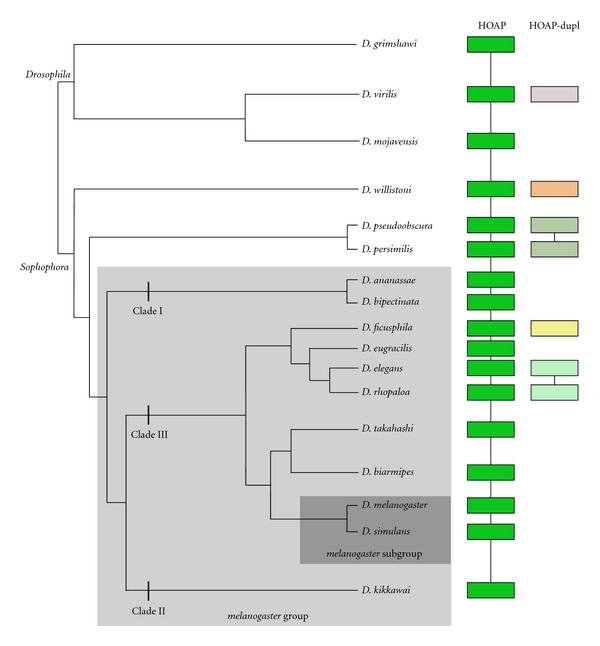
The HOAP protein family. The HOAP proteins were identified by tBLASTn search in Flybase (http://flybase.org/blast/) and are represented as rectangles. For the unannotated new sequenced genomes, BLAST analysis using 5 kbp upstream and downstream the *cav* and *cav-dupl* genes allowed to identify the orthologous region in the *melanogaster* genome. A same color code and a line connecting proteins indicate that the synteny block is conserved between the corresponding genes.

The highly dynamic repertoire of *K81*/*hiphop* genes reported here is remarkable and suggests that a constant evolutionary pressure is forcing this gene diversification (see below). One can wonder whether other telomere capping genes display a comparable level of evolutionary instability and, notably, those that are known to functionally interact with HipHop and K81. A great diversity of HP1 paralogs has already been documented in *Drosophila* [63], but the situation is complicated by the fact that HP1a is associated with several other important functions not related to telomere capping. In contrast, the other K81/HipHop partner HOAP is only required for telomere protection [48]. In *D. melanogaster*, the HOAP protein is encoded by a unique and essential gene named *caravaggio* (*cav*) [48]. HOAP is a fast evolving protein, which belongs to the *Drosophila* terminin complex of telomere proteins [46, 64]. This complex also contains two other proteins, Ver and Moi, which are also rapidly evolving as demonstrated by dN/dS analyses of their respective genes [46]. Interestingly, a recent study has reported the existence of three independent duplication of the *cav* gene outside the *melanogaster* group, in the *D. willistoni*, *D. virilis*, and *D. pseudoobscura/D. persimilis* lineages [65]. The presence of introns in these *cav* duplicates strongly suggests that these duplications occurred through a DNA-based mechanism. We found two additional independent duplication events in the recently released *melanogaster* group genomes (*D. ficusphila* and *D. elegans/D. rhopaloa* lineages) (Figure 2 and Table 2). Thus, although the presence of a syntenic *cav* gene in all *Drosophila* genomes sequenced so far indicates that this gene is probably more ancient than *hiphop*, it is also subjected to recurrent duplication events.

## 5. What Evolutionary Forces Drive the Diversification of Telomere Genes?

If the functional partitioning of these paralogs is well established by experimental and phylogenetic analyses, we now face the challenge of understanding the nature of the evolutionary force responsible for the birth of *K81*. Escape from MSCI and escape from DC cannot explain the case of *hiphop*/*K81* since both parental and daughter copies are autosomal, at least in the *melanogaster* subgroup. 

In the light of the duplication-degeneration-complementation classical model [61], the specialization of K81 in the capping of sperm telomeres as well as its restricted expression in the male germline are interpreted as the result of differential loss of function (i.e, subfunctionalization) of the duplicated copies [57–59]. In agreement with the DDC model, HipHop performs both somatic tissues and sperm-telomere capping in species without duplicates while in *D. melanogaster*, HipHop has lost its ability to protect chromosome ends in spermatids. Indeed, HipHop cannot replace K81 in complementation experiments. However, a simple subfunctionalization scenario does not predict the observed recurrent duplications of these capping genes that we have found here. A possibility is that the expression of a gene in testis increases the chance to get a testis-specific duplicate for mechanistic reasons (see the “out of testis” hypothesis in the introduction). 

The high gene turnover observed within the *hiphop*/*K81* gene family is more consistent with ongoing sexual conflicts, as recently proposed by Gallach and Betrán [13]. Their model states that a preexisting sexual conflict between different alleles of a gene could be solved by a gene duplication event and the acquisition of testis-specific expression of the duplicate. They propose that many testis-specific gene duplicates could have emerged under this scenario, including *K81*. This model implies that the ancestor gene plays an essential function in all cells (housekeeping gene), which is indeed the case of *hiphop*, as demonstrated by its associated lethal mutant phenotype and its critical protection of chromosome ends. In contrast, the duplicate gene *K81* is specifically expressed in the male germline and is first detected in spermatocytes. The K81 protein then decorates telomeres throughout spermiogenesis (postmeiotic stages of spermatogenesis) and after fertilization on paternal chromosomes [57, 58]. *hiphop* is actually also expressed in the male germline, but only in premeiotic cells [58]. Moreover, as previously mentioned, complementation analyses have demonstrated that HipHop and K81 have functionally diverged. Although this divergence could reflect an initial sexual conflict between different allelic variants of the ancestor protein, the Gallach and Betràn model states that acquisition of a testis-specific duplicate could also solve conflicting constraints on the expression of a ubiquitous parental gene. Indeed, genes that are specifically expressed in male germ cells are characterized by peculiar 5' regulatory elements and are often clustered in genome regions, suggesting the existence of higher order chromatin structure that favors transcription in spermatocytes or even in postmeiotic spermatids (reviewed in [2]). In this context, the existence of a duplicated copy could provide a more robust expression in the male germline than the ubiquitously expressed parental gene. This prediction could be experimentally tested by comparing the expression of *hiphop/K81*-like genes in species where a duplication has occurred or not. 

 These features fit with the possible existence of an initial sexual antagonism at the ancestor locus, which has been resolved by duplication followed by the specialization of the new copy. The fact that *hiphop* is actually expressed in the male germline is in apparent contradiction with this hypothesis. However, the critical difference between *hiphop* and *K81* is their differential expression in postmeiotic germ cells. Indeed, *K81* regulatory sequences drive robust and specific expression of K81 in spermatids, while the ubiquitously expressed HipHop is essentially excluded from these differentiating cells. 

Thus, the birth of *K81* may have removed this possible source of conflict at the ancestor locus. In this model, telomere capping genes that do not function in postmeiotic male germ cells are not expected to give rise to testis-specific duplicates. It would thus be interesting to investigate the distribution and function of other essential telomere capping genes in the male germline, such as *Ver* and *Moi, *that do not show any duplicate in the species analyzed in the present study (not shown). Interestingly, our phylogenetic analysis of the *cav* (HOAP) gene revealed a rather different diversification pattern. In contrast to *hiphop*, *cav* duplication events seem to occur only through a DNA-based mechanism, and we did not observe any obvious correlation between the *hiphop*/*K81* and the *cav* respective diversification patterns. *cav* is notably characterized by the presence of a fixed parental gene throughout the analyzed genomes, which is not the case for *hiphop*. Reis et al. [65] observed that the *D. willistoni cav-dup* is specifically (albeit weakly) expressed in males, but the other *cav* duplicates are expressed in both sexes. Thus, despite their apparent close functional relationship, these telomere genes are probably not subjected to the same evolutionary constraints. In addition, the functional status of *cav* in spermatids and sperm remains to be established. 

## 6. Concluding Remarks 

The molecular identification of the *K81* paternal effect gene about a decade ago was soon followed by the surprising observation that this essential male fertility gene in *D. melanogaster* was absent in the only other sequenced *Drosophila* genome available at that time (*D. pseudoobscura*) [55]. We now know that the acquisition of essential functions by recently evolved genes is not exceptional. A large-scale functional analysis of recently arisen genes in *Drosophila* revealed that most of them rapidly acquire essential developmental functions [66]. The functional characterization of new genes is invaluable to approach the intimacy of the evolutionary forces responsible for their origination and selection. Our phylogenetic analysis of the *hiphop*/*K81* gene family over twenty *Drosophila* species has revealed a highly dynamic pattern of gene gains and losses. Instead of our initial vision of a sporadic event specifically affecting the *melanogaster* subgroup, the *hiphop/K81* family is apparently subjected to a constant diversification. Future work should aim at determining if this diversification is compatible with the resolution of a sexual antagonism or with the “out of testis” hypothesis. 

## Figures and Tables

**Table 1:** hiphop and K81-like genes in *Drosophila*.

Species*\*gene	ID# or GI#	Orthologous region in *D. mel *	PTV/QFVH motif	Position of putative start codon	Position of stop codon
*D. mel\*hiphop	CG6874	*D.mel hiphop*	R R **PTV** -LDKQSMD		
*D. mel\*K81	CG14251	*D.mel K81*	RR **QFVH** LNREAMA		
*D. sim\*hiphop	GD14769	*D.mel hiphop*	RR **PTV** -LDKPSMD		
*D. sim\*K81	GD21311	*D.mel K81*	RR **QFVH** LNHQAMA		
*D. bia\*hiphop	358392949	*D.mel hiphop*	RR **PTVH** LNKEAMD	690387	689698
*D. bia\*K81	358402098	*D.mel K81*	RR **QFIH** LNKEAMD	2964671	2965297
*D. tak\*K81	343975433	*D.mel K81*	RR **QFVH** LNKEAMD	141804	141217
*D. tak\*hiphop-like	343974900	chro2L in fred gene	RR **QFVH** LNKEAMD	211517	212122
*D. rho\*hiphop	358405427	*D.mel hiphop*	RR **YVP** -LNKVAMD	33547	32867
*D. rho\*K81-like	358404732	chroX in Sh gene	RR **QFVH** LNKEAMD	683852	683265
*D. rho\*hiphop-K81-like-1	358405183	chroX Roc1a/CG13367	RR **FVA** -PNKEVMD	799350	800057
*D. ele\*hiphop	343972741	*D.mel hiphop*	RR **QVVH** PNKKAMD	ND	1725959
*D. ele\*K81-like	343972552	chroX in Sh gene	RR **QFVH** LNKNAMD	34447	35022
*D. ele\*hiphop-K81-like-1	343972719	chroX Roc1a/CG13367	RR **PTTI** LNKESMD	1005656	1006243
*D. eug\*hiphop	358409234	*D.mel hiphop*	RR **PVTH** LNKEAMD	677060	676191
*D. eug\*K81	358409002	*D.mel K81*	RR **QFVH** LNKEAME	154852	155409
*D. fic\*K81	343464569	*D.mel K81*	RR **SFVH** LNKEAMD	2599414	2600109
*D. fic\*hiphop-K81-like-1	343464682	chroX Roc1a/CG13367	RR **PTVH** LNKEAMD	461020	461505
*D. fic\*hiphop-K81-like-2	343464675	chro2L CG34163/zuc	RR **PALH** LNKEAMD	185420	184971
*D. kik\*hiphop-like	343973849	chro2L bsf/CR43344	RR **QFLV** PNKKVMD	92534	92040
*D. ana\*hiphop	GF10272	chro3L YT521-B/Drs	RR **PTII** LNKAVMD		
*D. bip\*hiphop	358403122	chro3L YT521-B/Drs	RR **QTVI** LNKAAMD	1284107	1283427
*D. pse\*hiphop	GA19922	*D.mel hiphop*	RR **QVVH** LNKTAMD		
*D. wil\*hiphop	GK12110	*D.mel hiphop*	RR **QIQ** -LTGPHLD		
*D. wil\*hiphop-like	GK15167	chro2L Or33c/Cry	RR **QVN** -RSGIDLD		
*D. moj\*hiphop-like	GI17239	chro2L CG13398	R R **QFVH** LNKDVMD		
*D. vir\*hiphop-like	GJ17998	chro2L CG13398	RR **QFVH** LNKDVMD		
*D. gri\*hiphop-like	GH13489	chro2L CG13398	RR **QFVS** LNKDVMD		

The *hiphop* and *K81*-like genes were identified by tBLASTn search in Flybase (http://flybase.org/blast/).

For each gene, the ID number, when available, is indicated. For species whose genome is not yet annotated, a GI number corresponding to the scaffold DNA sequence is indicated with the position in the scaffold of the putative start codon (first methionine in phase with the homolog protein identified) and the stop codon. ND: not determined.

The orthologous region in the *D. melanogaster* genome surrounding the *hiphop-K81*-like gene is indicated as follows: chromosome and neighbor genes. When two genes are indicated, the *hiphop-K81*-like gene is placed in between. D. mel hiphop and D. mel K81 means that the synteny block is conserved between the gene of interest and *hiphop* or *K81* from *D.  melanogaster*, respectively.

The PTV or QFVH motifs of the HipHop/K81 proteins as defined by Gao et al. [58] are highlighted in bold.

*D. mel: Drosophila melanogaster; D. sim: D. simulans; D. bia: D. biarmipes; D. tak: D. takahashi; D. rho: D. rhopaloa; D. ele: D. elegans; D. eug: D. eugracilis; D. fic: D. ficusphila; D. kik: D. kikkawai; D. ana: D. ananassae; D. bip: D. bipectinata; D. pse: D. pseudoobscura; D. wil: D. willistoni; D. moj: D. mojavensis; D. vir: D. virilis; D. gri: D. grimshawi*.

**Table 2:** *cav* and *cav*-like genes in *Drosophila*.

Species*\*gene	ID# or GI#	Orthologous region in *D. melanogaster *
*D. mel\*cav	CG6219	*D.mel cav*
*D. sim\*cav	GD21077	*D.mel cav*
*D. bia\*cav	358402078	*D.mel cav*
*D. tak\*cav	343975000	*D.mel cav*
*D. rho\*cav	358405209	*D.mel cav*
*D. rho\*cav-dupl	358407419	chro2R CG1441/CG1513
*D. ele\*cav	343972724	*D.mel cav*
*D. ele\*cav-dupl	343972624	chro2R CG1441/CG1513
*D. eug\*cav	358408974	*D.mel cav*
*D. fic\*cav	343464694	*D.mel cav*
*D. fic\*cav-dupl	343464518	chro3L Eip74EF/CG7510
*D. kik\*cav	343973540	*D.mel cav*
*D. ana\*cav	GF16116	*D.mel cav*
*D. bip\*hiphop	358402982	*D.mel cav*
*D. pse\*cav	GA27250	*D.mel cav*
*D. pse\*cav-dupl	GA26940	chro3R CG2218/CG15536
*D. per\*cav	GL23417	*D.mel cav*
*D. per\*cav-dupl	GL14051	chro3R CG2218/CG15536
*D. wil\*cav	GK11387	*D.mel cav*
*D. wil\*cav-dupl	GK24325	chro2L jhamt
*D. moj\*cav	GI24179	*D.mel cav*
*D. vir\*cav	GJ14215	*D.mel cav*
*D. vir\*cav-dupl	GJ17001	chroX Upf2
*D. gri\*cav	GH18668	*D.mel cav*

The *cav* genes and duplications in the 8 new sequenced *Drosophila* genomes were identified by tBLASTn search in Flybase (http://flybase.org/blast/). For these genes, a GI number corresponding to the scaffold DNA sequence is indicated. *cav* homologs and duplication in other species are identified with their ID number.

For each gene, the orthologous region in the *D. melanogaster* genome surrounding the identified *cav* homologous gene is indicated as follows: chromosome and neighbor genes. D. mel cav means that the synteny block is conserved between the gene of interest and *cav* from *Drosophila melanogaster*.

*D. mel: D. melanogaster; D. sim: D. simulans; D. bia: D. biarmipes; D. tak: D. takahashi; D. rho: D. rhopaloa; D. ele: D. elegans; D. eug: D. eugracilis; D. fic: D. ficusphila; D. kik: D. kikkawai; D. ana: D. ananassae; D. bip: D. bipectinata; D. pse: D. pseudoobscura; D. wil: D. willistoni; D. moj: D. mojavensis; D. vir: D. virilis; D. gri: D. grimshawi.*
